# Assessment of brain delivery of a model ABCB1/ABCG2 substrate in patients with non-contrast-enhancing brain tumors with positron emission tomography

**DOI:** 10.1186/s13550-019-0581-y

**Published:** 2019-12-12

**Authors:** Beatrix Wulkersdorfer, Martin Bauer, Rudolf Karch, Harald Stefanits, Cécile Philippe, Maria Weber, Thomas Czech, Marie-Claude Menet, Xavier Declèves, Johannes A. Hainfellner, Matthias Preusser, Marcus Hacker, Markus Zeitlinger, Markus Müller, Oliver Langer

**Affiliations:** 1 Department of Clinical Pharmacology, Medical University of Vienna, Vienna, Austria; 2 Centre for Medical Statistics, Informatics, and Intelligent Systems, Medical University of Vienna, Vienna, Austria; 3 Department of Neurosurgery, Medical University of Vienna, Vienna, Austria; 4 Division of Nuclear Medicine, Department of Biomedical Imaging and Image-guided Therapy, Medical University of Vienna, Vienna, Austria; 5 Inserm, U1144, Paris, France; 6 Université Paris Descartes, UMR-S 1144, Paris, France; 7 Université Paris Descartes, Sorbonne Paris Cité, Paris, France; 8 Institute of Neurology, Medical University Vienna, Vienna, Austria; 9 Division of Oncology, Department of Medicine I, Medical University of Vienna, Vienna, Austria; 10 Preclinical Molecular Imaging, AIT Austrian Institute of Technology GmbH, Seibersdorf, Austria

**Keywords:** Non-contrast-enhancing brain tumor, Blood–brain tumor barrier, PET, [^11^C]Tariquidar, P-glycoprotein, Breast cancer resistance protein

## Abstract

**Background:**

P-glycoprotein (ABCB1) and breast cancer resistance protein (ABCG2) are two efflux transporters expressed at the blood–brain barrier which effectively restrict the brain distribution of the majority of currently known anticancer drugs. High-grade brain tumors often possess a disrupted blood–brain tumor barrier (BBTB) leading to enhanced accumulation of magnetic resonance imaging contrast agents, and possibly anticancer drugs, as compared to normal brain. In contrast to high-grade brain tumors, considerably less information is available with respect to BBTB integrity in lower grade brain tumors.

**Materials and methods:**

We performed positron emission tomography imaging with the radiolabeled ABCB1 inhibitor [^11^C]tariquidar, a prototypical ABCB1/ABCG2 substrate, in seven patients with non-contrast -enhancing brain tumors (WHO grades I–III). In addition, ABCB1 and ABCG2 levels were determined in surgically resected tumor tissue of four patients using quantitative targeted absolute proteomics.

**Results:**

Brain distribution of [^11^C]tariquidar was found to be very low across the whole brain and not significantly different between tumor and tumor-free brain tissue. Only one patient showed a small area of enhanced [^11^C]tariquidar uptake within the brain tumor. ABCG2/ABCB1 ratios in surgically resected tumor tissue (1.4 ± 0.2) were comparable to previously reported ABCG2/ABCB1 ratios in isolated human micro-vessels (1.3), which suggested that no overexpression of ABCB1 or ABCG2 occurred in the investigated tumors.

**Conclusions:**

Our data suggest that the investigated brain tumors had an intact BBTB, which is impermeable to anticancer drugs, which are dual ABCB1/ABCG2 substrates. Therefore, effective drugs for antitumor treatment should have high passive permeability and lack ABCB1/ABCG2 substrate affinity.

**Trial registration:**

European Union Drug Regulating Authorities Clinical Trials Database (EUDRACT), 2011-004189-13. Registered on 23 February 2012, https://www.clinicaltrialsregister.eu/ctr-search/search?query=2011-004189-13.

## Introduction

Malignant brain tumors are considered as the most debilitating tumor types, mostly due to a bad quality of life, poor prognosis and limited therapeutic success [1]. In 2010, in the USA alone, more than 130,000 patients with primary malignant brain tumors were identified [1].

The updated World Health Organization (WHO) classification of tumors of the central nervous system not only considers histological but as of 2016 also molecular criteria to guarantee for a more refined diagnosis of the tumors with the main goal to optimize the treatment strategies for each individual patient [2]. Depending on the tumor type and localization, treatment recommendations comprise surgical removal, adjuvant radiotherapy and/or chemotherapy (e.g., temozolomide) [1, 3]. However, current treatment approaches have shown unsatisfactory outcomes. In particular, chemotherapy has failed to improve survival leading to a lethal course of the disease within 12–18 months in particular in high-grade brain tumors.

Sequencing studies have identified molecular alterations in brain tumors which may constitute promising targets for their treatment with molecularly targeted anticancer drugs [4]. However, small-molecule inhibitors of these pathways have not demonstrated significant therapeutic efficacy in the clinic [5]. This has been attributed to the inability of most of these drugs to cross the blood–brain barrier (BBB) and achieve therapeutically effective concentrations inside the brain. The BBB consists of brain capillary endothelial cells linked by tight junctions, which limit paracellular diffusion of drugs into the brain [6]. The protective function of the BBB is further enhanced by efflux transport proteins in the luminal membrane of brain capillary endothelial cells, i.e., P-glycoprotein (ABC subfamily B member 1, ABCB1) and breast cancer resistance protein (ABC subfamily G member 2, ABCG2), which were shown to work together in restricting the brain entry of dual ABCB1/ABCG2 substrate drugs [7–9]. The majority of currently known molecularly targeted anticancer drugs are ABCB1/ABCG2 substrates and show very limited brain distribution [8, 9]. ABCB1 and ABCG2 may also be overexpressed in the membrane of brain tumor cells, which may thus form a second barrier to the effective treatment of brain tumors [10–14].

There is strong evidence that significant BBB disruption, as evidenced by contrast enhancement in T1-weighted magnetic resonance imaging (MRI) sequences following intravenous administration of gadolinium-based contrast agents, occurs in primary high-grade brain tumors [14, 15]. However, this BBB disruption is mostly restricted to the central, necrotic areas of brain tumors and does not extend to regions distant from the tumor core, where infiltrative tumor cells remain protected by an intact BBB [14, 15]. Moreover, gadolinium-based contrast agents possess distinct physicochemical properties as compared with small-molecule anticancer drugs and may, therefore, not be representative of brain tumor delivery of such drugs. In contrast to high-grade brain tumors, considerably less information is available with respect to BBB permeability in lower grade brain tumors, which encompass a heterogeneous group of tumors that are clinically, histologically and molecularly diverse and often progress to high-grade tumors [14–16]. These types of brain tumors usually show no or only little contrast enhancement on MRI.

We have developed [^11^C]tariquidar as a prototypical dual ABCB1/ABCG2 substrate radiotracer for positron emission tomography (PET) imaging [17]. [^11^C]Tariquidar is derived from the third-generation ABCB1 inhibitor tariquidar [18]. It is a metabolically stable substrate of mouse and human ABCB1 and ABCG2 [17, 19] and shows very low brain distribution when ABCB1 and ABCG2 are functional and markedly increased brain uptake when ABCB1 and ABCG2 are pharmacologically inhibited or genetically knocked out [17]. As such, tariquidar closely resembles many molecularly targeted anticancer agents, which may be of interest for the treatment of brain tumors (e.g., gefitinib, erlotinib, dasatinib, imatinib and pictilisib) [20]. The aim of the present exploratory study was to investigate regional brain distribution of [^11^C]tariquidar in patients suffering from non-contrast-enhancing brain tumors (WHO grades I–III) by means of PET imaging. In addition, quantitative targeted absolute proteomics (QTAP) was applied to determine ABCB1 and ABCG2 levels in surgically resected tumor tissue.

## Materials and methods

All study-related procedures were performed at the Medical University of Vienna in accordance with the International Conference on Harmonization-Good Clinical Practice guidelines and the Declaration of Helsinki. Standard protocol approval was obtained from the Ethics Committee of the Medical University of Vienna and the national competent authority. The study was registered under the European Union Drug Regulating Authorities Clinical Trials Database (EUDRACT) number 2011-004189-13 (date of registration: February 23, 2012; https://www.clinicaltrialsregister.eu/ctr-search/search?query=2011-004189-13). Prior to study participation, all subjects gave oral and written consent.

### Study population

A total of seven patients (p01–p07, four female and three male, mean age of 44 years at the time of the PET scan, range 37–57 years) with an intrinsic, non-contrast-enhancing brain tumor and elected for neurosurgery were enrolled in this study. However, one patient denied surgical intervention after study inclusion. PET scans were performed between June 2014 and October 2015. Tumor entities were based on histopathological and molecular analyses and ranged from low-grade to high-grade brain tumors according to the 2016 WHO Classification of Tumors of the Central Nervous System [2]. Patient recruitment was performed in close cooperation with the Department of Neurosurgery at the Medical University Vienna.

### Radiotracer synthesis

[^11^C]Tariquidar was synthesized and formulated in sterile 0.9% (w/v) aqueous saline solution/ethanol (9/1, v/v) containing 0.7% (v/v) polysorbate-80 for intravenous injection into study participants as described previously [21]. Molar activity at the time of injection was 31 ± 8 GBq/μmol and radiochemical purity was 97 ± 1%.

### PET imaging protocol and general study procedures

Prior to surgical brain tissue removal, all patients included in this study were scheduled for a 60-min dynamic [^11^C]tariquidar PET scan acquired in three-dimensional mode using an Advance PET scanner (General Electric Medical System, Milwaukee, WI). On the study day, a venous catheter was placed in a cubital vein for radiotracer injection and an additional arterial catheter was applied for arterial blood sampling. Subjects were placed in supine position on the imaging bed with the head stabilized in a fixing mold attached to the imaging bed. A brief transmission scan (duration: 5 min) was performed for tissue attenuation of photons prior to the brain PET scan. Subsequently, [^11^C]tariquidar was injected intravenously over 20 s (injected activity: 371 ± 26 MBq, corresponding to 11 ± 3 μg of unlabeled tariquidar) while simultaneous dynamic PET imaging was accompanied by arterial blood sampling. Arterial blood samples were collected initially at intervals of 7 s during the first 3 min after radiotracer injection and further on at 3.5, 5, 10, 20, 30, 40 and 60 min after injection. Whole blood was centrifuged to obtain plasma. Selected plasma samples were analyzed with a previously described solid-phase extraction protocol for radiolabeled metabolites of [^11^C]tariquidar [21]. In brief, plasma (2–4 mL) was diluted with water (2 mL), spiked with unlabeled tariquidar (10 μL, 20 mg/mL in DMSO), acidified with 5 M aqueous hydrochloric acid (40 μL), and loaded on a Sep-Pak vac tC18 cartridge (Waters Corp.), which had been pre-activated with methanol (3 mL) and water (5 mL). The cartridge was first washed with water (5 mL) and then eluted with methanol (2 mL) followed by aqueous ammonium acetate buffer (0.2 M, pH 5.0, 1.5 mL). Radioactivity in all three fractions (plasma, water, and methanol/buffer) was quantified in a gamma counter. Radioactivity in the plasma and water fractions contained polar radiolabeled metabolites, whereas unchanged [^11^C]tariquidar was recovered in the methanol/buffer fraction.

### PET data analysis

T1-weighted (pre- and post-gadolinium enhanced) as well as T2-weighted FLAIR MRI data (Siemens Magnetom Trio, Tim System, Siemens Healthcare Diagnostics GmbH, Austria) were available from routine clinical examinations. Summation PET images and T2-weighted FLAIR MRIs were co-registered. The tumor and a contralateral tumor-free brain area, which were both of comparable size (Additional file 1: Table S1), were manually defined as volumes of interest (VOIs) using PMOD 3.6 imaging software (PMOD Technologies LLC, Zürich, Switzerland), whereby adjacent major blood vessels or ventricular structures were avoided. In one patient (p03), an additional VOI was outlined on the PET images, which corresponded visually to enhanced radioactivity uptake within the tumor (tumor PET enhanced). In addition, a gray matter VOI for normal brain tissue covering the brain hemisphere contralateral to the tumor was extracted for all patients using the Hammersmith n30r83 3D maximum probability atlas of the human brain [22] as described previously [19]. Time–activity curves (TACs) in units of kBq/mL were generated for the entire 60-min scanning period.

As the percentage of radiolabeled metabolites in arterial plasma samples was < 10% at all studied time points and as a previous study has shown that modeling outcome parameters were very similar in healthy volunteers for metabolite corrected and uncorrected input functions [21], no metabolite correction was applied to the arterial plasma input function in the present study. The area under the brain and plasma TACs (AUC) was calculated using Prism 8.0 software (Software, La Jolla, CA, USA). The ratio between the brain AUC and plasma AUC, designated as AUCR, was calculated as a parameter of radiotracer brain distribution [19]. In addition, Logan graphical analysis [19, 23] was performed in Microsoft Excel using the arterial plasma input function (not corrected for metabolites) to determine total distribution volume (*V*_T_), which equals the brain-to-plasma radioactivity ratio at steady state.

### Preparation of plasma membrane fraction from brain tumor samples

Plasma membrane fractions were isolated as described previously with minor modifications [24]. Frozen tumor tissues collected during neurosurgery of four patients (p01, p02, p03, p05) were thawed at +4°C, washed at least twice with isotonic buffer solution A (10 mM phosphate buffer pH 7.4, 0.1 M KCl) containing a protease inhibitor cocktail, minced into 1-mm pieces and homogenized using an Ultra-Turrax® (IKA®-Werke GmbH & Co. KG, Staufen, Germany) for 5 min at +4°C. The homogenates obtained were centrifuged at 10,800*g* for 15 min at +4°C and the supernatants were collected and ultracentrifuged at 100,000*g* for 60 min at +4°C. The plasma membrane fraction was obtained from the resulting pellet which was suspended in buffer B (20 mM Tris, pH 7.4, 0.25 M sucrose, 5.4 mM EDTA) containing protease inhibitor cocktail. The BCA protein assay kit (ThermoFisher Scientific, Villebon sur Yvette, France) was used for the total protein quantification.

### Protein digestion

Plasma membrane fractions were digested as described previously without modifications [25, 26]. Briefly, proteins were solubilized in denaturing buffer (7 M guanidine hydrochloride, 10 mM EDTA, 500 mM Tris, pH 8.5), reduced by DTT and alkylated by iodoacetamide. The alkylated proteins were precipitated with methanol–chloroform–water, resolubilized in 1.2 M urea and 0.1 M Tris, pH 8.5. Samples were first digested using rLysC endoprotease (enzyme:protein ratio = 1:50) for 3 h at room temperature. Then trypsin (enzyme:protein ratio = 1:100) and 0.05% (W/W) ProteaseMAX were added and samples were incubated at 37°C overnight. The stable isotope-labeled peptide mixture (750 fmol of each labeled peptide/50 μg of total protein) was added in trypsic digest before ultrahigh-performance liquid chromatography–tandem mass spectrometry (UHPLC–MS/MS) analysis.

### Protein quantification by UHPLC–MS/MS

ABCB1, ABCG2 and Na^+^/K^+^-ATPase proteins were quantified by the determination of the peptide concentration using UHPLC–MS/MS in multiplexed selected reaction monitoring (SRM) method. Each peptide analyzed was specific to each protein and was released after protein digestion by trypsin. The selected peptides were FYDPLAGK (human specific), VGTQFIR (human and mouse specific) [27], and AAVPDAVGK [28] for ABCB1, ABCG2 and Na^+^/K^+^-ATPase, respectively. Samples were injected into an Acquity UPLC® system (Waters, Manchester, UK), equipped with an Acquity UPLC BEH® C18 column (Peptide BEH® C18 Column, 300Å, 1.7 μm, 2.1 mm × 100 mm) supplied by Waters (Guyancourt, France). The mobile phase consisted of mixture of water (formic acid 0.1% (v/v)) and acetonitrile. It was operated with a flow rate of 0.3 mL/min in gradient mode. The total duration of analysis was 34 min. Data were recorded with a Waters Xevo® TQ-S mass spectrometer (Waters, Manchester, UK). Measurements were performed using positive electrospray ionization (ESI) with ion spray capillary voltage at 2.80 kV. Drying gas temperature was set to 650°C at a flow rate of 800 L/h. Detection was performed in multiplexed SRM mode using three or four transitions per native or labeled peptide and the quantification CV% between transitions was lower than 5%. Skyline® software [29] was used for the optimization of the specific transition parameters (i.e., collision energy (CE) and peak integration). The area ratios of light to labeled peptide were exported from Skyline® and quantification was performed from calibration curves using Microsoft Excel®.

### Statistical analysis

This study was exploratory; sample size was based on feasibility and not on power to test a statistical hypothesis. Differences in PET imaging outcome parameters between tumor and tumor-free brain tissue were assessed with a Wilcoxon matched-pairs signed rank test. To assess correlations, the Spearman's rank correlation coefficient (*ρ*) was calculated. A *p* value < 0.05 was considered statistically significant.

## Results

### Patient population

Table 1 summarizes demographic data of the enrolled brain tumor patients. Out of the seven included patients, three were diagnosed with diffuse astrocytic and oligodendroglial tumors, grade II or III (p02, p03 and p06, Table 1). Further, p03 and p06 had already undergone previous brain surgery in the past and were re-evaluated towards brain tumor progression by their responsible physicians. P07 refused neurosurgical intervention after inclusion and PET imaging. Therefore, histopathological entity was unknown. At the time of the PET scan, five out of seven patients received antiepileptic therapy for seizure prophylaxis (Additional file 1: Table S2). P01, p03 and p04 were treated with antidepressants. P02 received a statin and p07 was under treatment for hypertension. Further, p06 received thyroid hormone replacement therapy. One subject (p05) was free of any medication.

**Table 1:** Demographic data of enrolled brain tumor patients

	p01	p02	p03	p04	p05	p06	p07
Weight (kg)	78	65	110	62	83	60	108
Age at time of PET (years)	43	57	43	37	38	42	42
Sex	F	F	F	M	M	F	M
Time difference between PET and surgery (days)	7	10	22	48	39	6	No surgery
Neuropathological diagnosis	Diffuse glioma, IDH-wildtype and 1p/19q-codeleted, NEC (not elsewhere classified)	Oligodendroglioma, IDH-mutant and 1p/19q-codeleted	(Focally) anaplastic oligodendroglioma, IDH-mutant and 1p/19q-codeleted	Dysembryoplastic neuroepithelial tumor	Subependymoma	(Focally) anaplastic astrocytoma, IDH-mutant	N.a.
Localization	Left frontal/central	Right fronto-temporal	Right frontal	Left mesiotemporal	Right mesiotemporal	Right fronto-temporo-insular	Left mesiobasal temporal
WHO classification (grade)	II	II	IIIa	I	I	IIIa	N.a.

M, male; F, female; N.a., not available

^a^Pre-operated

### Imaging data

**Fig. 1:**
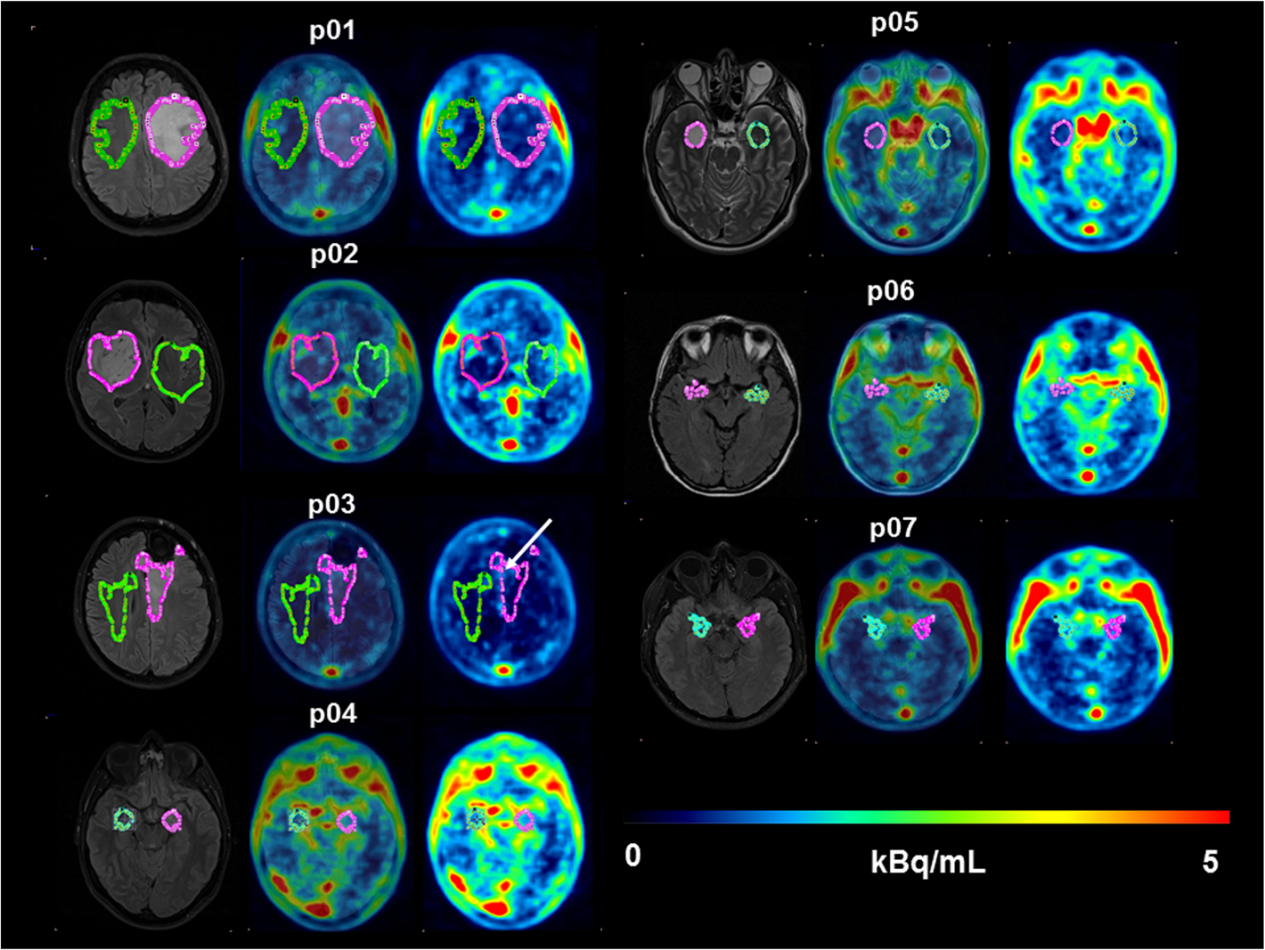
Transversal T2-weighted FLAIR MR images (left image), co-registered PET/T2-weighted FLAIR MR images (middle image) and [^11^C]tariquidar PET average images (0–60 min) (right image) in all patients. The contours for the tumor and contralateral tumor-free brain area are shown in pink and green colors, respectively. For p03, a tumor area with enhanced radioactivity uptake as compared with the rest of the tumor was visible (indicated by white arrow)

**Fig. 2:**
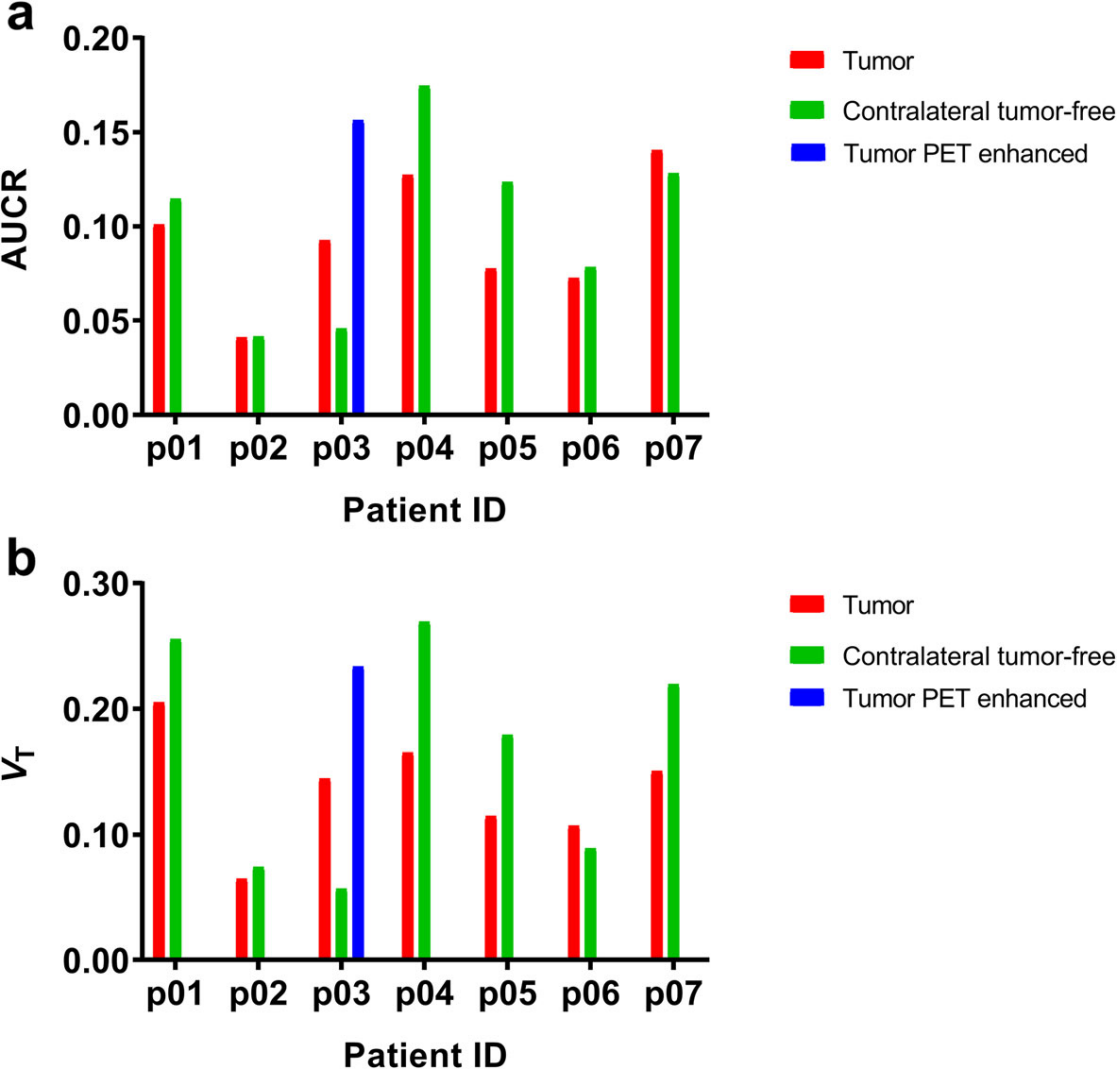
Outcome parameters for [^11^C]tariquidar brain distribution (**a** AUCR, **b** *V*_T_) in the tumor and contralateral tumor-free brain area (based on PMOD analysis). For p03, a tumor area with enhanced radioactivity uptake as compared with the rest of the tumor was visible (tumor PET enhanced)

Following intravenous injection of [^11^C]tariquidar, only a very low amount of radiolabeled metabolites was detected in plasma for the duration of the PET scan. At 60 min after radiotracer injection, 91.1 ± 3.1% of total radioactivity in plasma was in the form of unchanged [^11^C]tariquidar. Brain uptake of radioactivity was very low. Tumors were best visualized by T2-weighted FLAIR MRI with a median tumor volume of 12.0 cm^3^ (range 4.2–81.8 cm^3^) (Additional file 1: Table S1). None of the tumors showed appreciable gadolinium-enhanced areas on T1-weighted MRIs (not shown). In Fig. 1, T2-weighted FLAIR MR images, co-registered PET/T2-weighted FLAIR MR images and PET images are shown for all patients. In the PET images, no major visual differences in radioactivity distribution between tumor and tumor-free brain areas were evident (Fig. 1). However, in one patient (p03), a small area of enhanced radioactivity uptake (1.6 cm^3^) was observed within the tumor volume (44.9 cm^3^) (Fig. 1). As outcome parameters of [^11^C]tariquidar brain distribution, we determined AUCR and *V*_T_ [19], which are shown in Fig. 2 (based on the VOIs defined with PMOD). Normal brain gray matter *V*_T_ values from the entire brain hemisphere contralateral to the tumor (not shown in Fig. 2) were higher than those in contralateral tumor-free brain area containing both gray and white matter (0.38 ± 0.26 versus 0.16 ± 0.09). There was a good correlation between *V*_T_ values and AUCR values (ρ = 0.8762; *p* < 0.0001, not shown). AUCR and *V*_T_ values were very low and rather variable among patients, both in tumor and contralateral tumor-free brain areas. No significant differences in AUCR and *V*_T_ values between tumor and tumor-free areas were found (Fig. 2). In p03, the small area of enhanced radioactivity uptake within the tumor had higher AUCR and *V*_T_ values than the entire tumor and normal brain tissue.

### Tissue levels of ABCB1 and ABCG2

In four subjects (p01, p02, p03 and p05), surgically resected tumor tissue was analyzed for ABCB1 and ABCG2 levels with QTAP (Table 2). For the other two patients, not enough material was available for this analysis. As surgical specimens were only available as frozen tissue samples, the isolation of brain capillary micro-vessels was not feasible, so that the measured ABCB1 and ABCG2 levels represent an average value of membrane fractions of all cell types present in the sample. Mean ABCB1 and ABCG2 levels were 0.8 ± 0.1 and 1.2 ± 0.3 fmol/μg protein, respectively.

**Table 2:** Absolute ABCB1, ABCG2 and ATPase levels in plasma membrane fractions of surgically removed tumor tissue determined with quantitatively targeted absolute proteomics

	Total protein used for digestion (μg) (***n***)	ABCB1 (fmol/μg) (CV%)	ABCG2 (fmol/μg) (CV%)	ATPase (fmol/μg) (CV%)
p01	50 (1)	0.97 (<5%)	1.53 (<5%)	178.3 (<5%)
p02	50 (4)	0.79 (2.7 %)	0.85 (4.4 %)	257.1 (0.6%)
p03a	25 (1)	0.69 (<5%)	0.94 (<5%)	60.1 (<5%)
p03a	50 (3)	0.94 (6.4 %)	1.4 (8.2 %)	164.6 (7.1 %)
p05	40 (1)	0.64 (<5%)	1.10 (<5%)	83.3 (<5%)

For lower limits of quantification, see [27]

%CV, % coefficient of variation: *n* = digestion replicate, each digested sample was injected three times. Quantification CV% <5% is the variation between transitions and results from three injections (for the three samples digested once and injected three times)

^a^Two different samples collected during surgery

## Discussion

In this exploratory study, we used PET imaging to assess regional brain delivery of [^11^C]tariquidar as a small-molecule model ABCB1/ABCG2 substrate in patients with non-contrast-enhancing brain tumors. The main finding of our study was that brain delivery of [^11^C]tariquidar was comparably low in tumor and tumor-free brain tissue, which suggested that ABCB1/ABCG2 transport activity was sufficiently intact in tumor tissue to restrict brain entry of anticancer drugs which are dual ABCB1/ABCG2 substrates.

The blood–brain tumor barrier (BBTB) is formed by the capillaries supplying brain tumors. Depending on the tumor type and size, the BBTB may substantially differ from the BBB [30]. While low-grade brain tumors possess continuous non-fenestrated capillaries, which resemble normal brain capillaries, high-grade brain tumors often possess leaky, fenestrated vessels [31], as manifested by increased permeability to MRI contrast agents [32]. There is evidence that efflux transporters localized in the BBB can also be found in endothelial cells forming the BBTB [33, 34]. MRI contrast agents are hydrophilic, gadolinium-containing complexes which are believed to cross the BBTB via the paracellular route through fenestrated capillaries. On the other hand, molecularly targeted anticancer drugs are small, lipophilic molecules which mainly cross the BBB via the transcellular route and which are, in most cases, subject to efflux transport by ABCB1/ABCG2. Even in the presence of a disrupted BBTB with fenestrated capillaries, endothelial cells may have sufficient ABCB1/ABCG2 transport capacity to limit tumor distribution of such drugs [34, 35].

A limited number of previous studies have determined brain tumor concentrations of anticancer drugs [36–40]. Three studies assessed intratumoral concentrations of protein kinase inhibitors (gefitinib, imatinib and lapatinib) in surgically resected tumor tissue of glioblastoma patients and found very variable tumor concentrations of these agents, which in part exceeded the corresponding plasma concentrations, which pointed to an increased permeability relative to normal brain [36, 39, 40]. Blakely et al used intraoperative microdialysis to measure the intratumoral pharmacokinetics of methotrexate in patients with recurrent gliomas and found considerably higher drug concentrations in contrast-enhancing regions of the tumor as compared with non-enhancing tissue [38]. Finally, Brown et al. performed PET with the radiolabeled focal adhesion kinase inhibitor [^11^C]GSK2256098 in eight patients with recurrent glioblastoma [37]. Brain uptake (*V*_T_) of [^11^C]GSK2256098 was found to be very low, but approximately two times higher in tumor tissue as compared with surrounding T2 enhancing areas and normal brain. All these data supported a focal disruption of the BBTB in high-grade gliomas, which led to enhanced brain distribution of small-molecule drugs, which were subject to ABCB1 and/or ABCG2 efflux transport.

As opposed to these previous studies, we examined in the present study patients with non-contrast enhancing, low- to high-grade brain tumors and used a radiolabeled model ABCB1/ABCG2 substrate instead of a drug which is used for treatment of tumors. Previous experiments showed that [^11^C]tariquidar had very low brain uptake in wild-type, *Abcb1a/b*^*(-/-)*^ and *Abcg2*^*(-/-)*^ mice, but approximately sixfold higher brain uptake in triple knockout *Abcb1a/b*^*(-/-)*^*Abcg2*^*(-/-)*^ mice [17], which was in line with the typical behavior of a dual ABCB1/ABCG2 substrate [7]. In healthy human volunteers, brain uptake of [^11^C]tariquidar was very low, but significantly increased in carriers of the *ABCG2* single-nucleotide polymorphism c.421C>A following ABCB1 inhibition [19]. These data suggested that brain distribution of [^11^C]tariquidar is dependent on ABCB1/ABCG2 transport activity in rodents and humans. This is also true for the majority of currently known molecularly targeted kinase inhibitors, which have been proposed for the treatment of brain tumors [20, 41]. In fact, [^11^C]tariquidar behaved very similar in rodents and humans in terms of its brain distribution as [^11^C]erlotinib [42, 43], a radiolabeled epidermal growth factor receptor (EGFR) targeted tyrosine kinase inhibitor, which is also a dual ABCB1/ABCG2 substrate and which failed in a phase II trial in patients with glioblastoma multiforme [44]. In our study, brain distribution of [^11^C]tariquidar was found to be very low throughout the brain including tumor tissue, except for one patient with a grade III brain tumor, who had undergone previous surgery and in whom a small area of increased radiotracer uptake was observed within the tumor (Fig. 1). This suggested that the investigated tumors received their blood supply through intact capillaries, which efficiently restrict brain distribution of small-molecule ABCB1/ABCG2 substrates. The standard treatment for malignant brain tumors is temozolomide. While temozolomide is believed to penetrate the BBB relatively well, a recent study has shown that brain entry of temozolomide is increased in the absence of ABCB1 and ABCG2 activity in mice which translated into an improved antitumor response in experimental intracranial tumor models [45]. It can, therefore, be expected that brain delivery of temozolomide in tumor patients is also restricted, at least to some extent, by ABCB1/ABCG2 activity.

In comparison to our previous study in healthy volunteers [19], mean *V*_T_ of [^11^C]tariquidar in normal brain tissue was approximately threefold lower (0.16 ± 0.09 in this study versus 0.43 ± 0.10 in healthy volunteers). The percentage of unmetabolized [^11^C]tariquidar in plasma at the end of the PET scan was comparable in tumor patients and in healthy volunteers (91.1 ± 3.1% in tumor patients versus 89.7 ± 3.6% in healthy volunteers), which rules out an effect of the concomitant medication taken by the tumor patients (Additional file 2: Table S2) on radiotracer metabolism as an explanation for the observed differences in radiotracer brain distribution. One possible explanation for the observed differences may be differences in the applied VOIs. In healthy volunteers, whole brain gray matter was analyzed [19], while in tumor patients, the position of the applied (contralateral) VOI for normal brain tissue depended on the localization of the brain tumor and contained both gray and white matter. This assumption is supported by additional analysis of the data in brain tumor patients with the same methodology as employed in reference [19], which provided a normal brain gray matter *V*_T_ value of 0.38 ± 0.26 in patients. It is noteworthy that distribution of [^11^C]tariquidar to normal brain tissue displayed a considerably higher inter-individual variability in tumor patients as compared with healthy volunteers.

Tariquidar is a third-generation ABCB1 inhibitor which has undergone clinical development as a multidrug resistance reversal agent in patients with systemic tumors [18]. However, its clinical development has been stopped due to lack of efficacy in tumor patients. In the past decade, tariquidar has been investigated as a potential inhibitor of ABCB1-mediated efflux transport at the human BBB. PET imaging studies in healthy volunteers revealed up to fivefold increases in brain distribution of the radiolabeled model ABCB1 substrates (*R*)-[^11^C]verapamil and [^11^C]*N*-desmethyl-loperamide following tariquidar administration [46, 47]. Pharmacological inhibition of efflux transporters at the BBB has also been proposed for a more effective treatment of brain tumors with anticancer drugs, for which brain distribution is limited by ABCB1/ABCG2-mediated efflux transport [14, 30]. In this context, it has been suggested that ABCB1 inhibition may additionally improve access of anticancer drugs to tumor cells which overexpress ABCB1 in their cell membranes [13, 14]. However, to achieve effective ABCB1 inhibition in brain tumor cells, an ABCB1 inhibitor would first need to cross the BBTB. The data presented in this work show that tariquidar very poorly penetrates the BBTB and may, therefore, not be effective to overcome ABCB1-mediated multidrug resistance of brain tumors.

To examine ABCB1 and ABCG2 levels, we performed QTAP on surgically resected brain tissue samples of four patients included in this study (Table 2). QTAP allows for obtaining absolute levels of proteins in the brain and has been applied to measure ABCB1 and ABCG2 levels in human brain micro-vessels [48]. In our study, micro-vessels could not be isolated; therefore, the measured ABCB1 and ABCG2 levels represented membrane-bound transporters of all cell types present in the sample (e.g., micro-vessels, glia cells, neurons and tumor cells). Accordingly, mean ABCB1 levels (0.8 ± 0.1 fmol/μg protein) and ABCG2 levels (1.2 ± 0.3 fmol/μg protein) were approximately seven times lower than those previously reported in isolated human brain capillary micro-vessels (ABCB1: 6.1 ± 1.7 fmol/μg protein, ABCG2: 8.1 ± 2.3 fmol/μg protein) [48]. However, ABCG2/ABCB1 ratios in our samples were comparable (1.4 ± 0.2, range: 1.1-1.7) to previously reported values from isolated human brain micro-vessels (1.3) [48], which suggested that no major ABCB1 or ABCG2 overexpression occurred in the investigated tumors.

## Conclusion

We found very low brain delivery of the model ABCB1/ABCG2 substrate [^11^C]tariquidar in patients with non-contrast-enhancing brain tumors without significant differences between tumor and tumor-free brain tissue. This supports the presence of an intact BBTB, which is impermeable to small-molecule ABCB1/ABCG2 substrates. This potentially applies to a range of small-molecule kinase inhibitors, which are dual ABCB1/ABCG2 substrates and which are discussed as potential treatment for brain tumors. The best strategy for an effective treatment of brain tumors may thus be the development of drugs with good passive permeability which are not subject to ABCB1/ABCG2-mediated efflux transport at the BBTB [41, 49, 50].

## Supplementary information

**Additional file 1: Table S1.** Volumes of interest (cm^3^) for analyzed brain tissue. **Table S2.** List of continuous medication at the time of the PET scan.

## Data Availability

The datasets generated during and/or analyzed during the current study are available from the corresponding author on reasonable request.

## Abbreviations

ABCB1: ABC subfamily B member 1 also known as P-glycoprotein
ABCG2: ABC subfamily G member 2 also known as breast cancer resistance protein
AUC: Area under the curve
AUCR: AUC ratio
BBB: Blood–brain barrier
BBTB: Blood–brain tumor barrier
MRI: magnetic resonance imaging
PET: Positron emission tomography
QTAP: Quantitative targeted absolute proteomics
SUV: Standardized uptake value
TAC: Time–activity curve
*V*_T_: Total distribution volume
VOI: Volume of interest
WHO: World Health Organization
