# Supplementary material for: Assessment of brain delivery of a model ABCB1/ABCG2 substrate in patients with non-contrast-enhancing brain tumors with positron emission tomography
Source: EJNMMI Res. 2019 Dec 12;9:110. doi: 10.1186/s13550-019-0581-y (PMC6908538; doi:10.1186/s13550-019-0581-y)
Supplement: Supplementary file 1 — Additional file 1: Table S1. Volumes of interest (cm3) for analyzed brain tissue. Table S2. List of continuous medication at the time of the PET scan. [file 13550_2019_581_MOESM1_ESM.docx]

**ELECTRONIC SUPPLEMENTARY MATERIAL**

**Assessment of brain delivery of a model ABCB1/ABCG2 substrate in patients with non-contrast enhancing brain tumors with positron emission tomography**

Beatrix Wulkersdorfer^1^, Martin Bauer^1^, Rudolf Karch^2^, Harald Stefanits^3^, Cécile Philippe^4^, Maria Weber^1^, Thomas Czech^3^, Marie-Claude Menet^5^, Xavier Declèves^5^, Johannes A Hainfellner^6^, Matthias Preusser^7^, Marcus Hacker^4^, Markus Zeitlinger^1^, Markus Müller^1^, Oliver Langer^1,4,8,*^

^1^ Department of Clinical Pharmacology, Medical University of Vienna, Vienna, Austria

^2^ Centre for Medical Statistics, Informatics, and Intelligent Systems, Medical University of Vienna, Vienna, Austria

^3^ Department of Neurosurgery, Medical University of Vienna, Vienna, Austria

^4^ Division of Nuclear Medicine, Department of Biomedical Imaging and Image-guided Therapy, Medical University of Vienna, Vienna, Austria

^5^ Inserm, U1144, Paris, France; Université Paris Descartes, UMR-S 1144, Paris, France; Université Paris Descartes, Sorbonne Paris Cité, Paris, France

^6^ Institute of Neurology, Medical University Vienna, Vienna, Austria

^7^ Division of Oncology, Department of Medicine I, Medical University of Vienna, Vienna, Austria

^8^ Preclinical Molecular Imaging, AIT Austrian Institute of Technology GmbH, Seibersdorf, Austria

Correspondence: [oliver.langer@meduniwien.ac.at](mailto:oliver.langer@meduniwien.ac.at)

**Table S1.** Volumes of interest (cm^3^) for analyzed brain tissue

|  | **Tumor** | **Contralateral tumor-free** | **Tumor PET enhanced** |
| --- | --- | --- | --- |
| p01 | 57.4 | 53.7 | - |
| p02 | 81.8 | 74.0 | - |
| p03 | 44.9 | 45.9 | 1.6 |
| p04 | 4.6 | 4.5 | - |
| p05 | 4.2 | 4.0 | - |
| p06 | 12.0 | 11.2 | - |
| p07 | 7.3 | 7.2 | - |

**Table S2.** List of continuous medication at the time of the PET scan

|  | **Trade Name** | **Substance Name** | **Dose (mg)** | **Frequency** | **Daily Dose (mg)** |
| --- | --- | --- | --- | --- | --- |
| p01 | Trittico retard | Trazodon hydrochloride | 150 | 1x/d | 150 |
|  | Levetiracetam | Levetiracetam | 500 | 2x/d | 1000 |
| p02 | Crestor | Rosuvastatin | 20 | 1x/d | 20 |
|  | Lamotrigin | Lamotrigin | 50 | 2x/d | 100 |
| p03 | Trittico retard | Trazodon hydrochloride | 150 | 1x/d | 50 |
|  | Levetiracetam | Levetiracetam | 1000 | 2x/d | 2000 |
|  | Cymbalta | Duloxetin | 60 | 1x/d | 60 |
|  | Frisium | Clobazam | 10 | 1x/d | 5 |
| p04 | Cipralex | Escitalopram | 10 | 1x/d | 10 |
|  | Levebon | Levetiracetam | 500 | 2x/d | 1000 |
| p05 | - | - | - | - | - |
| p06 | Thyrex | Levothyroxin natrium | 100 | 1x/d | 100 |
|  | Levetiracetam UCB | Levetiracetam | 1500 | 2x/d | 3000 |
| p07 | Blopress | Candesartan cilexetil | 16 | 1x/d | 16 |

d, day
